# Retraction Note: Dual loading miR-218 mimics and Temozolomide using AuCOOH@FA-CS drug delivery system: promising targeted antitumor drug delivery system with sequential release functions

**DOI:** 10.1186/s13046-021-02064-9

**Published:** 2021-08-11

**Authors:** Li Fan, Qian Yang, Jiali Tan, Youbei Qiao, Qiaofeng Wang, Jingya He, Hong Wu, Yongsheng Zhang

**Affiliations:** 1 Department of Pharmaceutical Analysis, School of Pharmacy, Fourth Military Medical University, Xian, 710032 Shaanxi China; 2 Institute of Materia Medica, School of Pharmacy, The Fourth Military Medical University, Xian, 710032 Shaanxi China; 3 Department of Orthodontics, Guanghua School of Stomatology, Hospital of Stomatology, Sun Yat-sen University & Guangdong Provincial Key Laboratory of Stomatology, Guangzhou, 510055 China; 4 Department of Administrative, Tangdu hosipital, Fourth Military Medical University, Xian, 710038 Shaanxi China; 5 Department of Pharmaceutical Chemistry, School of Pharmacy Fourth Military Medical University, Xian, 710032 Shaanxi China

**Retraction Note: J Exp Clin Cancer Res 34, 106 (2015)**

**https://doi.org/10.1186/s13046-015-0216-8**

The Editor-in-Chief has retracted this article. Concerns were raised regarding Figure 2, specifically:

- ▪ the top two features in the upper panel appear to be identical to each other, and appear to overlap with the top feature in the lower panel.
- ▪ the bottom feature in the upper panel appears to partially overlap with the bottom feature in the lower panel.
- ▪ the backgrounds in the upper and lower panel appear to be identical.

The Editor-in-Chief therefore no longer has confidence in the reliability of the data reported in the article.

Li Fan, Quin Yang, Jiali Tan, and Hong Wu agree to this retraction. Youbei Qiao, Qiaofeng Wang, Jingya He, and Yongsheng Zhang have not responded to any correspondence from the editor/publisher about this retraction.

